# Sterilization by Adaptive Immunity of a Conditionally Persistent Mutant of Mycobacterium tuberculosis

**DOI:** 10.1128/mBio.02391-20

**Published:** 2021-01-19

**Authors:** Catherine Vilchèze, Steven A. Porcelli, John Chan, William R. Jacobs

**Affiliations:** a Department of Microbiology and Immunology, Albert Einstein College of Medicine, Bronx, New York, USA; Sequella, Inc.

**Keywords:** conditional persistence, *Mycobacterium tuberculosis*, sterilizing immunity

## Abstract

Mycobacterium tuberculosis (*Mtb*), the causative agent of tuberculosis, can enter into a persistent state that confers resistance to antibacterial agents. Many observations suggest that persistent M. tuberculosis cells also evade the antimycobacterial immune mechanisms, thereby reducing the effectiveness of the current tuberculosis vaccine. Understanding the factors that contribute to persistence may enable the rational design of vaccines that stimulate effective immune killing mechanisms against persister cells. Independent mutations targeting the methionine and arginine biosynthetic pathways are bactericidal for M. tuberculosis in mice. However, in this study, we discovered that the addition of leucine and pantothenate auxotrophy altered the bactericidality of methionine auxotrophy. Whereas the leucine/pantothenate/methionine auxotrophic M. tuberculosis strain H37Rv Δ*leuCD* Δ*panCD* Δ*metA* was eliminated in immunocompetent mice, this strain persisted in multiple organs of immunodeficient *Rag1^−/−^* mice for at least a year. In contrast, the leucine/pantothenate/arginine auxotroph H37Rv Δ*leuCD* Δ*panCD* Δ*argB* was eliminated in both immunocompetent and immunodeficient *Rag1^−/−^* mice. Our results showed that leucine and pantothenate starvation metabolically blocked the sterilization mechanisms of methionine starvation but not those of arginine starvation. These triple-auxotrophic strains should be invaluable tools for unravelling the bacterial and host factors that enable persistence and for vaccine development studies to assess the efficacy of vaccines that boost immune recognition of M. tuberculosis in the persistent state. The sterilization of the Δ*leuCD* Δ*panCD* Δ*metA* auxotroph in immunocompetent mice, but not in mice lacking an adaptive immune response, could provide a new system for studying the antimycobacterial killing mechanisms of adaptive immunity.

## Abstract (precis)

The bacterial pathogen Mycobacterium tuberculosis can enter into a persistent state in which M. tuberculosis can evade host immunity, thereby reducing the effectiveness of current tuberculosis vaccines. Understanding the factors that contribute to persistence would enable the rational design of vaccines effective against persisters.

## OBSERVATION

Tuberculosis (TB) remains a significant global health problem despite the availability and widespread use of chemotherapy and of bacille Calmette-Guerin (BCG) vaccination. Our previous research has demonstrated that the effectiveness of chemotherapy is hampered by the ability of Mycobacterium tuberculosis (*Mtb*) to enter into a persistent state (1, 2). We hypothesize that persistence may also be a factor in the limited effectiveness of BCG or most other approaches to TB vaccination. As part of our goal to develop better TB vaccines, we have been exploring how various host and bacterial factors modulate the immune response and bacterial survival. Understanding such factors will aid in developing improved TB vaccines and in establishing the correlates of protection in humans that will aid vaccine design and selection. BCG, because of its relative attenuation in virulence, has been used as the challenge strain in humans for testing the efficacy of novel vaccine candidates (3, 4). However, the ability of BCG to cause disseminated infections in humans (5–7) and its lack of several important immune targets, such as those contained in the *esx-1* region of difference (RD1), limit its usefulness (8–11). The ideal strain for TB vaccine challenge studies in humans would be safe, susceptible to immune killing, and contain the appropriate immunological targets.

We propose that auxotrophic mutants of M. tuberculosis represent alternative human *in vivo* challenge organisms as they are safer than BCG, while retaining the RD1 region and other genetic loci deleted in BCG (10–12). In auxotrophic mutants, a specific biosynthetic gene or pathway is mutated, resulting in a conditional mutation in which the mutant will grow only when the product of that biosynthetic pathway is supplied. We have observed that infection with different auxotrophic mutants of M. tuberculosis can have different outcomes *in vivo*, with some mutants inducing bacteriostatic host responses and persisting in host tissues, whereas others induce bactericidal responses in which the bacteria are eliminated, resulting in sterilization of infected tissues. For example, M. tuberculosis leucine/pantothenate double auxotrophs persist in immunocompetent and immunocompromised mice (13, 14), whereas the M. tuberculosis methionine H37Rv Δ*metA* (15) and arginine H37Rv Δ*argB* (16) auxotrophs are sterilized. These observations led us to hypothesize that the induction of a persister phenotype or a sterilization phenotype by nutrient limitations is governed by the nature of the targeted biosynthetic pathway.

**FIG 1:**
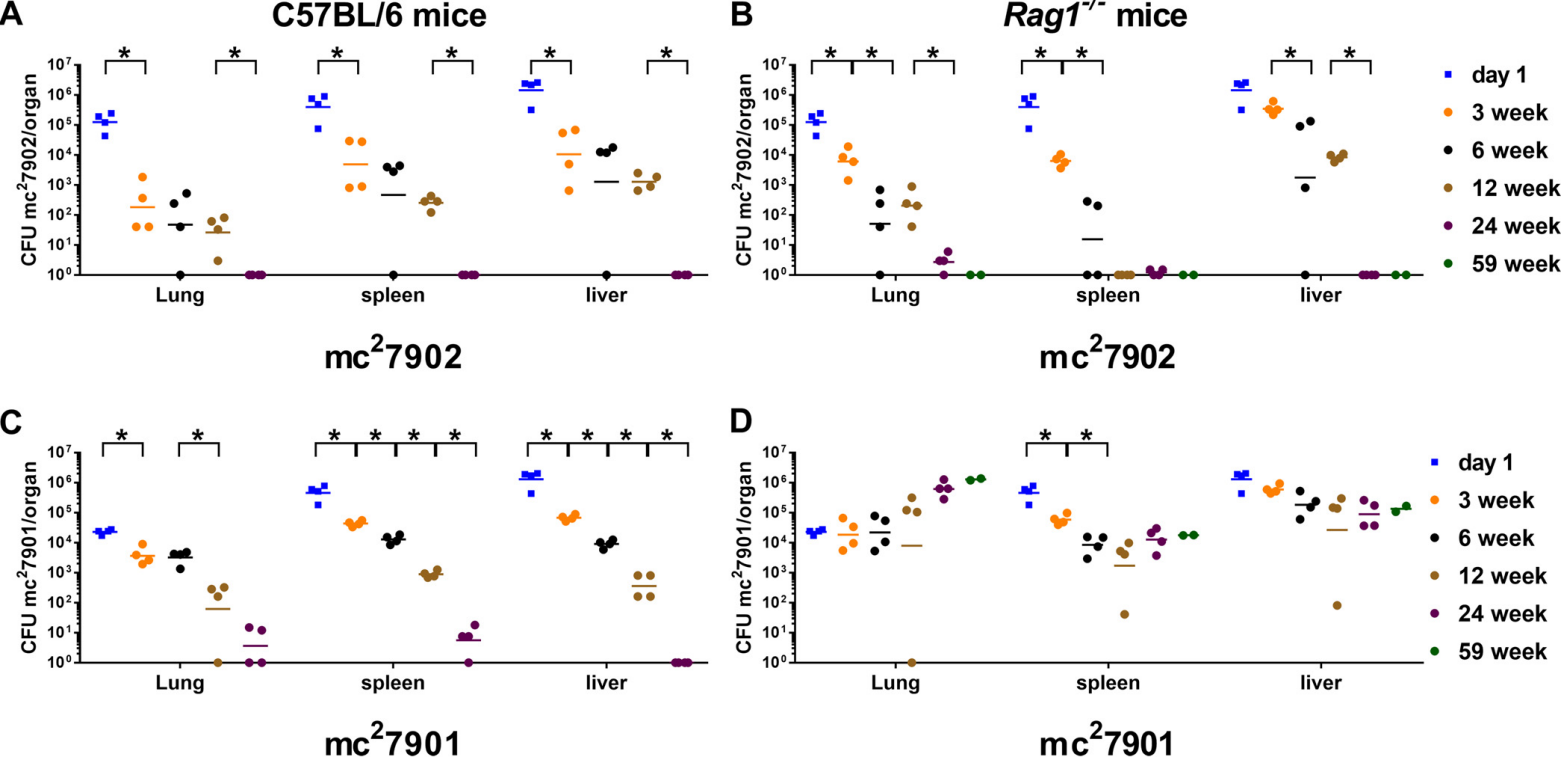
Strain mc^2^7901 persists in immunocompromised *Rag1^−/−^* mice but not in immunocompetent mice. C57BL/6 mice (A and C) and *Rag1^−/−^* mice (B and D) were infected via intravenous injection with 3.8 × 10^6^ CFU of mc^2^7902 (H37Rv Δ*leuCD* Δ*panCD* Δ*argB*) (A and B) or 3.0 × 10^6^ CFU of mc^2^7901 (H37Rv Δ*leuCD* Δ*panCD* Δ*metA*) (C and D). At the indicated times, four mice per group were euthanized, and lungs, livers, and spleens were homogenized to determine burden of M. tuberculosis. The 59-week time point is composed of only two *Rag1^−/−^* mice per group. Data that are statistically significant are marked with an asterisk (*P* < 0.05).

Previously, we mutated the leucine/pantothenate auxotroph H37Rv Δ*leuCD* Δ*panCD* (mc^2^6206) by introducing a third auxotrophy, i.e., methionine or arginine auxotrophy (17). We hypothesized that deleting either *metA* or *argB* from mc^2^6206, which undergoes bacteriostasis in mice (13), would render the resulting strains susceptible to rapid sterilization in mice, given that when made independently the *metA* and *argB* mutations were bactericidal during mouse infections. To test this hypothesis, immunocompetent C57BL/6 mice and immunodeficient *Rag1^−/−^* mice, which lack adaptive immunity, were infected intravenously with the leucine/pantothenate/methionine auxotroph H37Rv Δ*leuCD* Δ*panCD* Δ*metA* (mc^2^7901) or the leucine/pantothenate/arginine auxotroph H37Rv Δ*leuCD* Δ*panCD* Δ*argB* (mc^2^7902). Lungs, livers, and spleens were harvested at 3, 6, 12, 24, and 59 weeks postinjection, and bacterial burdens were determined. As expected for a triple auxotroph, the leucine/pantothenate/arginine auxotroph mc^2^7902 was eliminated by 24 weeks in the livers, lungs, and spleens of both immunocompetent and immunodeficient mice (Fig. 1A and B). Similarly, the leucine/pantothenate/methionine auxotroph mc^2^7901 was eliminated by 24 weeks in immunocompetent mice (Fig. 1C). However, mc^2^7901 persisted in all three organs in immunodeficient mice during the entire 59 weeks of the experiment even though initial dissemination to the lungs (day 1) was nearly a log unit lower for mc^2^7901 than for mc^2^7902 (Fig. 1C and D). A small increase in the bacterial burden in the lungs of mc^2^7901-infected immunodeficient mice was observed over time, but this was not statistically significant. In livers and spleens, titers in mc^2^7901-infected immunodeficient mice decreased for the first 6 to12 weeks of infection, although at a lower rate than observed for mc^2^7902, before plateauing. The ability of mc^2^7901 to persist in immunodeficient mice but not in immunocompetent mice suggests that some component of the adaptive immune response is able to kill mc^2^7901. This unexpected observation revealed a conditional system of persistence that renders M. tuberculosis susceptible to killing by an adaptive immune response.

The profound difference in the persistence phenotypes between the two strains in the immunocompromised host was surprising. Persistence is the capacity of a subpopulation of bacterial cells to survive a sterilizing process (18–20). Persisters do not acquire a mutation but rather turn on a genetic program to survive killing by a drug or other assault. The study of the survival of these triple-auxotrophic M. tuberculosis strains *in vivo* highlights a few important observations. First, while the sterilization mediated by methionine starvation of our Δ*metA* mutant can be reversed by combining the *metA* metabolic block with blocks in the leucine and pantothenate biosynthetic pathways, the arginine starvation sterilization pathway is not affected by the leucine and pantothenate auxotrophy. Second, the persistent leucine/pantothenate/methionine auxotroph mc^2^7901 can remain protected from the innate immune system for over 1 year and cause no clinically recognizable disease in mice lacking adaptive immunity. Last, the sterilization of mc^2^7901 in immunocompetent mice reveals the presence of an M. tuberculosis-killing mechanism that is dependent on a functional adaptive immune response.

As of today, BCG remains the only licensed TB vaccine. While numerous new TB vaccines have been developed (21), an animal model that reliably delineates the correlates of protection in humans remains to be established. We propose that the triple auxotroph mc^2^7901, a biosafety level 2-safe laboratory strain (13, 17), which is markedly attenuated *in vivo*, could provide a safe test strain for assessing the efficacies of TB vaccines in humans. Since M. tuberculosis is not a recipient of conjugation in nature and the Δ*leuCD*, Δ*panCD*, and Δ*metA* deletions were shown not to be reversible or suppressible (14, 16, 22), these sets of auxotrophies are unlikely to be repaired *in vivo*. Indeed, only two independent mutations in M. tuberculosis are required by the World Health Organization to qualify a strain as a vaccine (23). A suitable human infection model of TB must be safe, with limited replication *in vivo* and easily and reproducibly quantifiable *in vivo* or *ex vivo*. Various markers for monitoring strain elimination, such as the NanoLuc gene or its optimized version Antares (24, 25), could be incorporated into mc^2^7901 to enable expeditious evaluation of vaccine efficacy after intradermal challenges as described for BCG in the human vaccine model (3, 4). Moreover, the use of *rag1^−/−^* mice and mc^2^7901 provides an attractive model for dissecting the components of the immune response that can elicit protection. Last, recently isolated clinical strains, which might be more relevant for vaccine assessment, can be used as substrates to generate safe triple auxotrophs as challenge strains for human vaccination models, through targeting the leucine, pantothenate, and methionine/arginine metabolic pathways by specialized transduction (13). The utility of these safe, multiple-auxotrophic strains is multifold. They provide a platform that enables direct, cost-saving, and expeditious evaluation of novel anti-TB vaccines in humans and can define correlates of protection in TB. Such strains also constitute a set of invaluable tools for unraveling the mechanisms underlying M. tuberculosis persistence, as well as for the discovery of drugs that can eliminate persisters and deliver sterilizing immunity in humans.

### Experimental procedures. (i) Bacterial strains and reagents.

The M. tuberculosis strains mc^2^7901and mc^2^7902 were obtained from laboratory stocks. The strains were grown in 30-ml square bottles containing 5 ml Middlebrook 7H9 (Difco, Sparks, MD) supplemented with 10% (vol/vol) oleic acid-albumin-dextrose-catalase (OADC; Difco), 0.2% (vol/vol) glycerol, d-pantothenate (24 mg/liter), l-leucine (50 mg/liter), l-methionine (50 mg/liter), l-arginine (200 mg/liter), and 0.05% (vol/vol) tyloxapol at 37°C with shaking. Plating was done using Middlebrook 7H10 (Difco) supplemented with 10% (vol/vol) OADC, 0.2% (vol/vol) glycerol, d-pantothenate (24 mg/liter), l-leucine (50 mg/liter), l-methionine (50 mg/liter), and l-arginine (200 mg/liter), and the plates were incubated at 37°C for 4 to 8 weeks. Media and chemicals were obtained from Sigma (St. Louis, MO) or ThermoFisher Scientific (Waltham, MA).

### (ii) Mouse infection.

Female C57BL/6 mice were obtained from Envigo (Somerset, NJ). Female *Rag1^−/−^* mice (6 to 8 weeks old) were bred in-house. Animal protocol AUP20171114 was approved by the Einstein Animal Institute, which is accredited by the American Association for the Use of Laboratory Animals (DHEW publication no. [NIH] 78-23, revised 1978), and accepts as mandatory the NIH “Principles for the Use of Animals.” The mc^2^7901 and mc^2^7902 strains were grown to mid-log phase (optical density at 600 nm [OD_600_] of ∼0.6 to 0.8), pelleted by centrifugation, and washed twice with Dulbecco’s phosphate-buffered saline (DPBS) containing 0.05% tyloxapol (DPBS-tyloxapol). Cells were resuspended in DPBS-tyloxapol, sonicated twice for 10 s, and diluted in DPBS-tyloxapol to a concentration of 1.5 × 10^7^ CFU/ml. Mice were infected via tail vein injection (0.2 ml injected). At day 1 and at weeks 3, 6, 12, 24, and 59, mice were euthanized. The lungs, spleens, and livers were homogenized in DPBS to determine CFU per organ. Serial dilutions of the lysates in DPBS were plated on fully supplemented Middlebrook 7H10 plates (see above), and the lowest dilutions were also plated on Middlebrook 7H10 plates without amino acid supplements. The plates were incubated at 37°C for 4 to 8 weeks to obtain CFU counts.

### (iii) Statistics.

Differences between groups were analyzed by an unpaired, nonparametric Mann-Whitney test using GraphPad Prism 7.05 (San Diego, CA).
